# He/She/They - gender inclusivity in developing and using health-related questionnaires: a scoping review

**DOI:** 10.1007/s11136-024-03765-2

**Published:** 2024-08-29

**Authors:** Desiree Scott, Sarah Derrett, Valentina Prevolnik Rupel, Jennifer Jelsma, Gagan Gurung, Georgina Yaa Oduro, Cassie Withey-Rila

**Affiliations:** 1 Department of Health and Rehabilitation Sciences, University of Cape Town, Cape Town, South Africa; 2 Ngāi Tahu Māori Health Research Unit, Division of Health Sciences, University of Otago, Dunedin, New Zealand; 3 Ministry of Health, Ljubljana, Slovenia; 4 Department of General Practice and Rural Health, University of Otago, Dunedin, New Zealand; 5 Health New Zealand, Te Whatu Ora, National Public Health Service, Te Waipounamu, New Zealand; 6 Department of Sociology and Anthropology, University of Cape Coast, Cape Coast, Ghana; 7 Department of Medicine, University of Otago, Christchurch, New Zealand

**Keywords:** Gender inclusive, Language, Research, Health care, Advisory group

## Abstract

**Purpose:**

To ensure the recognition and participation of all potential respondents in health research, surveys and care, including LGBTQIA + broadly, and trans people, specifically, the use of inclusive language should be considered. This scoping review aimed to identify and describe strategies considered for gender inclusivity in development and use of health questionnaires and Patient Reported Outcomes Measures (PROMs).

**Methods:**

A systematic search of peer reviewed publications between January 2000 and September 2022 was conducted in Scopus, ProQuest Central, Ovid Medline (PubMed and EBSCO). Two reviewers independently screened identified publications titles and abstracts, followed by full text screening and data extraction from eligible articles.

**Results:**

The search of over 5000 publications, retained 18; most acknowledged gaps in representation and advocated for gender-inclusive language. Eight articles discussed exclusion from health care and health research for gender minority groups due to the use of gendered language. Improved reliability, validity and response rates were associated with the use of gender-neutral language in seven articles. Only one article reported finding irritation among cisgender males when non-binary gender response options were used. One paper, focussing on instruments for Rheumatoid Arthritis, discussed gaps in representation if diverse gender identities were not considered when developing PROMs.

**Conclusion:**

This scoping review points to the importance of adopting gender-inclusive language in health questionnaires and surveys to reduce the risk of excluding gender minority groups. Despite finding very few specific examples of how others have used gender-inclusive language in health questionnaires, many strengths of gender-inclusive language usage were identified.

## Abstract (plain language summary)

People from the LGBTQIA + communities are often excluded from health research and from completing heath surveys as they are not able to identify with the questions about male or female genders. This scoping review aimed to examine the literature for any strategies developers of health questionnaires may have come up with to ensure inclusion of all demographic groups. Most of the identified literature highlights the importance of adopting gender-inclusive language in health questionnaires to reduce the risk of excluding gender minority groups. Despite many strengths of gender-inclusive language usage being identified, only a few specific examples were identified of how others have used gender-inclusive language in health questionnaires.

## Introduction

There have been increased calls for diverse gender identities to be acknowledged to ensure appropriate recognition and inclusion of all people in health and social care systems and research contexts [1–7]. The World Health Organization (WHO) considers how ‘gender’ differs from ‘sex’. Sex they note refers “…to the different biological and physiological characteristics of females, males and intersex persons, such as chromosomes, hormones and reproductive organs”, whereas gender refers to the “characteristics of women, men, girls and boys that are socially constructed” [8]. The WHO further describes how gender identity “…refers to a person’s deeply felt, internal and individual experience of gender, which may or may not correspond to the person’s physiology or designated sex at birth” [8]. While the WHO, and others, have adopted this descriptive dichotomy, a multidimensional measure of sex and gender ascertaining biological sex and social gender does risk considering sex as being an ‘objective truth’ and gender as ‘socially constructed’. It is important to note that both sex and gender are equally relevant as determinants of health [9, 10].

Sociodemographic characteristics are often collected in health questionnaires, forms, and surveys, and it can be unclear whether researchers are asking about ‘sex’ or ‘gender’ [11]. Additionally, the choices available to respondents are often binary or dichotomous such as male/female, man/woman and boy/girl, but gender is not binary – a point acknowledged by many Indigenous societies (e.g. Takatāpui; part of the whānau (extended family kin group) Māori, New Zealand) [12]. Clearly, binary ‘male/female’ gender categories do not provide gender response options for all people (e.g. those who identify as transgender, genderfluid, agender, and gender diverse).

Problems arise when questionnaires and surveys only use the binary gender categories (e.g. male or female). These include: misclassification and ‘invisibility’ of non-binary gender communities (e.g. if non-binary gender respondents are obliged to select one of only two gender options to be able to proceed with the questionnaire), reduced representativeness of populations in study samples (e.g. if people decline to participate in studies as a consequence of not being able to identify appropriate genders relevant to them), and discrimination (e.g. non-binary respondents perceiving they are not valued or recognised) [11, 13]. Indeed, developing questionnaires inclusive of all genders has been identified as an issue of ethical importance for researchers [14]. It has also been noted that when researchers use binary language they are contributing to the ongoing construction of gender as binary [13].

Addressing binary gender issues in health questionnaires and surveys is undoubtedly complex. In many languages, including English and Spanish, gender bias is introduced when the masculine form is used to describe a mixed gender group (e.g. policemen) [15–17]. Gender bias may be avoided by providing both male and female options in combination, such as in s/he, but this does not include the wide diversity of genders [15]. Grammatically gendered languages, including the Romance languages (French, Spanish or Italian), German and Czech, assign gender to personal nouns as well as to inanimate nouns [17, 18]. Other languages, such as Norwegian and Dutch, do not use grammatical gender, only inanimate nouns are gendered allowing for the use of gender neutral options [19]. Languages that do not separate the genders in personal nouns or pronouns (e.g. Estonian, Finnish, Turkish, Hungarian, and Māori) are genderless languages, which do not require specific strategies to become gender neutral or inclusive [19]. English, Swedish and Norwegian are considered natural gendered languages as they do not make use of grammatical gender but do use personal pronouns to distinguish between male and female (he and she). Swedish has introduced a gender neutral third person pronoun, ‘hen’ [20]. There is a difference between gender neutrality and gender inclusivity. ‘Gender neutral’ refers to language that does not use any gender categories, whereas ‘gender-inclusive’ language acknowledges and includes the many gender identities beyond binary male/female categories [15]. In some societies or communities, there is a move to a more gender-inclusive options such as (in English) using the singular ‘their’ rather than ‘his/her’ [19, 21]. To promote inclusion of all people, gender inclusivity should be incorporated into the development of all health surveys [22].

The EuroQoL Group (DS, SD, VR and JJ are EuroQol Group members) develops and maintains instruments to assess health, including a range of health-related quality of life instruments (e.g. the EQ-5D-5 L, EQ-5D-3 L and EQ-5D-Y) [23]. These instruments are widely used throughout the world by governments, health research companies, and researchers; currently, EuroQol instruments have been translated into over 200 languages. EuroQol instruments ask people to report their health, typically usually using self-complete (paper or digital) or interviewer administered questionnaires. If self-completion is not possible, for example because the person is too unwell to self-complete a questionnaire or respond to an interviewer’s questions, then proxy (where the proxy is asked to rate the health of the other person) versions are available. The English language EuroQol proxy versions, which act as the ‘source’ versions for translation into other languages, currently use gendered language such as he/she and himself/herself (e.g. “Would you say s/he has no problem walking about”) [23]. This potentially excludes populations such as the LGBTQIA + community broadly, and trans people, specifically, from participating as they would not identify with “she” or “he”. Subsequently this could negatively affect the representativeness of the study sample, for example, if the general population was the target group [1, 7, 24–26].

We sought to understand the approaches used by others to address gender inclusivity in the development of other questionnaires before making suggestions to alter to the current, rigorously developed, EuroQol instruments.

Objective.

The broad aim of this scoping review was to identify, describe, and understand strategies considered by others for gender inclusivity in development and use of data collection tools and measures with a particular focus on health-related questionnaires (i.e. instruments, measures and/or survey tools) applicable to the general population and populations with a health condition.

The review intended to answer the following research questions with reference to questionnaires which considered the effect of gendered language, especially health-related questionnaires:

1. What rationale is provided to support the development and use of gender-inclusive language in different countries?
2. What strategies and processes have been used to develop and use gender-inclusive language in different language groups?
3. What specific examples are reported of gender-inclusive language being applied in health outcome measures?
4. What are the reported facilitators and barriers faced by developers and users while considering gender inclusivity in developing and using health-related questionnaires and how have these been addressed?

## Method

The six-stage methodological framework described by Arksey and O’Malley’s [27] was used: identifying the research question, searching for relevant articles, selecting articles, charting the data, collating, summarizing, and reporting the results, and consulting with stakeholders to inform or validate findings. The scoping review was guided by an advisory group which included members from diverse communities with expertise in gender research and HRQoL. The group assisted in defining the specific research questions, advised on search terms to be included and inclusion and exclusion criteria, synthesis of the findings of the scoping review and drawing logical conclusions.

### Data sources and research strategy

The initial search for relevant published literature began in September 2022. With the assistance of an experienced librarian and input from the advisory group, the following search terms were used: “gender inclusi*” OR “gender-inclusi*” OR “non-gendered” OR “nongendered” OR “non-binary” OR “gender neutral” OR “gender-fair language” OR “gender equity language” OR “gender-neutral pronouns” OR “lgbt*” OR “bisexual*” OR “lesbian*” OR “gay*” OR “transgender*” OR “intersex” AND “data collection” OR “instrument*” OR “measure*” OR “tool*” OR “questionnaire*” OR “survey*” OR “standard*” OR “census” OR “scale*”.

AND

“language” OR “terms” OR “term.

Searches in electronic databases were conducted in Scopus, ProQuest Central, Ovid Medline which includes PubMed and EBSCO Academic Search Complete. The snowball method was used to identify any additional articles from reference lists in included articles.

### Eligibility

The following eligibility criteria were developed and applied in an initial test search and then revised following an initial search and input from the advisory group (Table 1).

**Table 1:** Test and final criteria for inclusion and exclusion criteria

Test criteria	Final criteria
Inclusion criteria	Inclusion criteria
1. Empirical articles which explicitly considered gender inclusivity in questionnaires (such as health measures/instruments/questionnaires/surveys/census questions/healthcare implementation/intervention studies/attitudes to gendered language).	1. Publications which explicitly considered the language/terms of gender inclusivity in questionnaires (such as health measures/instruments/questionnaires/ surveys/census questions/healthcare implementation/intervention studies/attitudes to gendered language).
2. Published in peer reviewed academic journals.	2. Full text publications in peer reviewed academic journals in English and/or German, Nepali, or Slovenian, and English translations of other languages.
3. Publications from national/international organisations (e.g. WHO, WB or IMF or EU) identified from the bibliographic searching of included published literature.	3. Publications from national/international organisations or unions (e.g. WHO, WB or IMF or EU) identified from the bibliographic search of the included published literature.
4. Published from 1 January 2000 to 21 September 2022.	4. Published from I January 2000 to 21 September 2022.
5. Opportunistic discovery of useful articles in the references of an excluded main article.	5. Opportunistic discovery of useful publications in the references of eligible articles.
Exclusion criteria	Exclusion criteria
1. Studies or articles reporting sex and/or gender as demographic variables but which did not describe or discuss gender inclusivity.	1. Publications reporting sex and/or gender as demographic variables but not describing or discussing the language of gender inclusivity.
2. Grey literature (apart from that identified in hand searching of bibliographies mentioned in eligibility criteria #3 above).	2. Grey literature (apart from that identified in hand searching of bibliographies mentioned in eligibility criteria #3 above).
3. Research published outside eligible date range.	3. Publications outside eligible date range.
4. Questionnaires used for other than health and census purposes.	4. Data collection tools used for other than health research and census purposes.

Many articles in the test search were found to focus on health professionals’ care of LGBTQIA+/trans people in hospital which was not the intended focus of this scoping review. These criteria were revised to also include “language and /or terms of gender inclusivity”. Having researchers in the group who understand German, Nepali, or Slovenian, enabled articles in these languages to be included.

## Results

### Study selection

Following the search of the five selected data bases, 8215 publications were initially identified and imported into EndNote [27]. After 2719 duplicates were removed, title and abstract screening occurred for 5496 publications. An initial screening of the first 5% (*n* = 275) was completed independently by two reviewers (DS and GG) who each recorded the publications they thought would fit the inclusion criteria. Following a meeting between the two reviewers, no substantive disagreements were found and a total of 14 publications were agreed on. The reviewers then screened the remainder independently applying the inclusion criteria, and there were no further changes. A further 561 duplicates were found. The reviewers met again to compare their selection and discuss reconciliations; there were 12 publications where the reviewers were not in agreement. Two other reviewers (SD and VR) independently counter reviewed these articles; ultimately, six of the 12 were retained. A total of 60 publications were retained for full text review.

Next, the 60 full text articles were independently reviewed by two reviewers (DS and SD), using the inclusion criteria. Additionally the references of the 60 articles were searched for any relevant publications which fell within the inclusion criteria or referred to national/international organisations policies or guidelines on gender-inclusive language but had not been identified in the search. This process identified a further 10 possible publications resulting in the full text review of 70 articles for eligibility. Initially, 15 articles were reviewed independently and compared for agreement. No disputes arose, so reviewing of the remaining 55 continued independently by DS and SD. Following discussion between the two reviewers, 11 full text articles were then counter reviewed by a third reviewer (VR) to confirm they met the inclusion criteria.

**Fig. 1:**
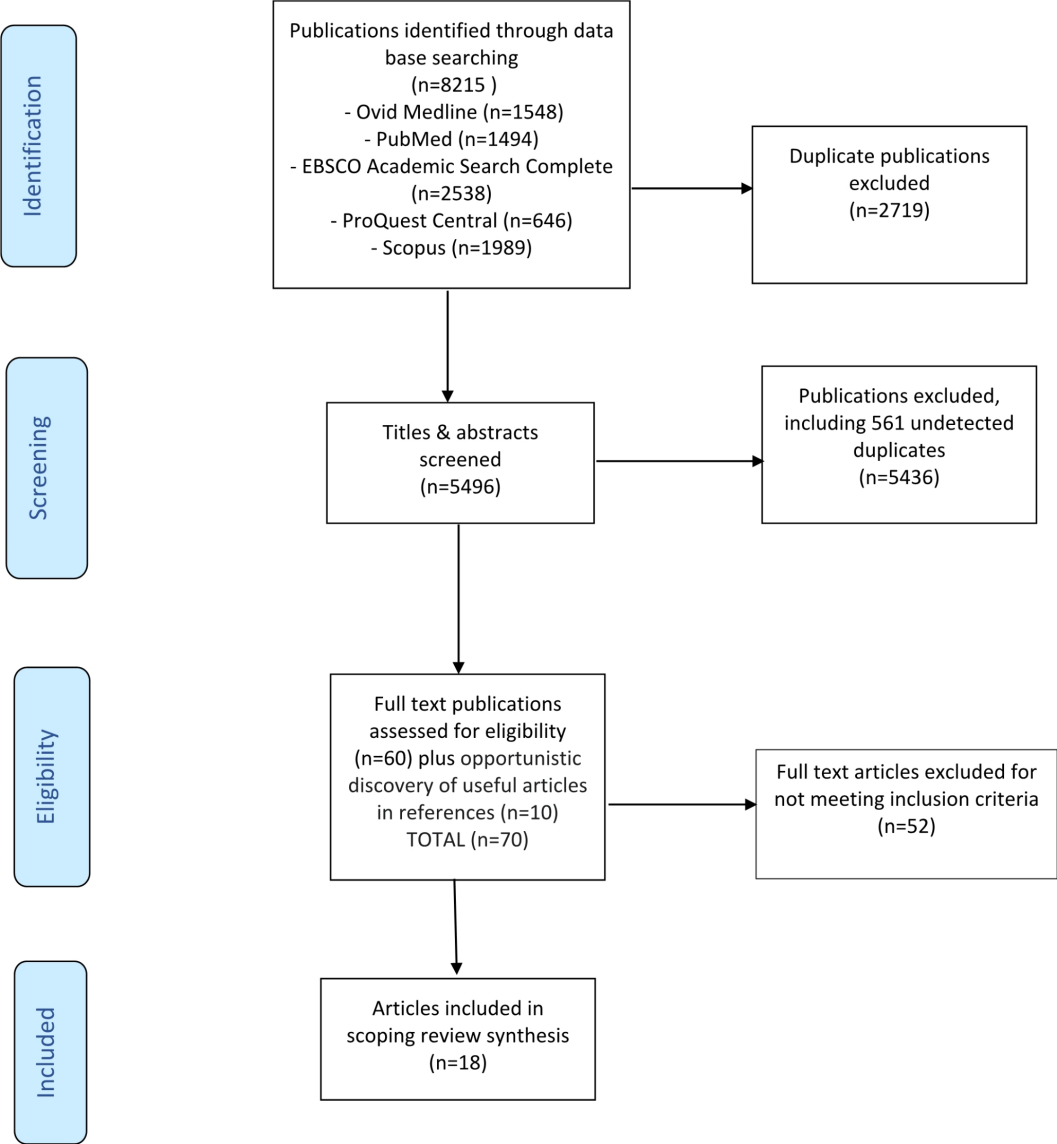
PRISMA Flow Chart

. The exclusion of 52 publications which did not meet the inclusion criteria resulted in a final set of 18 articles (17 from the original 60 and 1 from the additional 10) being included for data extraction (Fig. 1).

### Data extraction and charting

One of the researchers (DS) extracted relevant data from the articles and entered into an Excel Spreadsheet according to Arksey and O’Malley’s methodological framework [27] (Table 2). This included the following characteristics of the articles considering language/terms of gender inclusivity in data collection tools:

- Title, year of publication, author(s), journal.
- Study location (country and context).
- Aims of the study.
- Methods: Study design, participants and number, outcome measures used.
- Findings in relation to gender inclusivity.
- Limitations in relation to this scoping review.

**Table 2:** Characteristics of articles considering language/terms of gender inclusivity in questionnaires

#	Source		Population			Methodology		Findings	
	Title (Year) Journal	Authors	Study location (country)	Aim of Study	Participants (gender as reported in article)	Study design	Outcome measure used	Important results	Any limitations in relation to our scoping review
1	Sex and gender considerations in implementation interventions to promote shared decision making: A secondary analysis of a Cochrane systematic review (2020) *PLoS ONE* [28]	Adisso, Évèhouénou Lionel; Zomahoun, Hervé Tchala Vignon; Gogovor, Amédé; Légaré, France	Not applicable - a secondary analysis of a Cochrane systematic review	An assessment of sex and gender terminology in Shared Decision Making (SDM) in intervention studies.[4]	87 intervention studies	A secondary analyses of systematic reviews using the adapted the Preferred Reporting Items for Systematic Reviews and Meta-Analyses (PRISMA).	Measured frequencies of the use of sex- and gender-related terms according to three criteria for correct use of sex and gender terms according to the CIHR and NIH categories. These were: “non-binary use”, male, female or intersex, “use of appropriate categories” to describe sex and gender and “non-interchangeable use of sex and gender” if sex terms and gender terms were mentioned and indiscriminately used to describe either sex or gender of participants.	None of the intervention studies met all criteria for correct use of the terms for reporting on sex and gender. Sex and gender were used interchangeably or were used as binary variables only, creating confusion about to whom the interventions were applicable. This resulted in non-binary individuals feeling excluded by the health system and being invisible in research results.	While the focus was on measuring sex and gender in SDM intervention studies, the significant negative impact of not considering correct terminology to include all sex and gender types, on health and health communications about health, would be relevant to our outcome measures.
2	Assessing the impact of gender-neutral language on disclosure of sexual violence. (2012) *Psychology of Violence* [4]	Anthony, Elizabeth R; Cook, Sarah L.	USA A southeastern university.	An experimental investigation into effect of survey language on disclosure of victimization and perpetration experiences, when adopting gender-neutral language in the Sexual Experiences Survey (SESR).	258 female and 190 male college students	Between-subjects experimental design, randomly assigning participants to either a gender-neutral language or gender-specific language condition.	Revised Sexual Experiences Survey (SESR) and a modified SESR with truly gender-neutral language.	Article does focus on the language of gender inclusivity in surveys - although specifically in relation to sexual violence surveys. It did not find any statistically significant effects of language on disclosure. However, “…various researchers have demonstrated significant effects of survey wording on response rates (Fisher, Cullen, & Turner, 2000; Hamby & Koss, 2003), illustrating the importance of survey language for self-report measures”.	Sample was mostly heterosexual, so the impact of language when asking sexual minority populations is not fully known.
3	Misgendering as epistemic injustice: a Queer STS approach. (2021) *Las Torres De Lucca* [29]	Argyriou, Konstantinos	Not applicable - report from Resources from Science, Technology and Society (STS) Studies.	The paper discusses the forms of misgendering observed in the completion of surveys, questionnaires and official documents and the analysis of the Gender-Fair Language Model to overcome exclusion of Transgender and Gender Non-Conforming (TGNC) people.	Resources from Queer Science, Technology and Society (STS) Studies.	Discussion of reports by the author.	Resources from Science, Technology and Society (STS) Studies.	“Misgendering is the use of improper pronouns and grammatical genders to refer to people who belong to the Transgender and Gender Non-Conforming (TGNC) umbrella. Language plays a central role in misgendering.” Most biomedical survey data only allow for males and females, excluding TGNC identities from health-related studies. Suggested the introduction of Gender Fair Language to administrative and official accounts of gender, to ensure inclusion of all people.	Focusses on how reporting of gender on health surveys, is mostly binary, thereby excluding TGNC people identities. Gender Fair Language should be employed to overcome this, but not specific to any particular health measure.
4	Validation studies of rheumatoid arthritis patient-reported outcome measures in populations at risk for inequity: A systematic review and analysis using the OMERACT summary of measurement properties equity table. (2022) *Seminars in Arthritis and Rheumatism* [25]	Barnabe, Cheryl; Wattiaux, Aimée; Petkovic, Jennifer; Beaton, Dorcas; Shea, Beverley; Greer-Smith, Regina; Humphreys, Jenny; Bartels, Christie; Tugwell, Peter; Umaefulam, Valerie	Not applicable - systematic review	Aimed to identify measurement property studies for PROMs in patients with RA who were at risk for inequity by various factors including sex and gender identity.	Rheumatoid Arthritis (RA) patients	Included experimental, observational, and qualitative studies.	PROM instruments: Pain VAS; DAS 28; ACR 20; Patient Global Assessment; EQ-5D; PROMIS.	In this systematic review there were “…notably, no studies examining the impacts of diversity in gender identity and expression nor sexual orientation on PROMs”. The study highlighted important gaps in patient representation. New outcome measures being developed for research purposes and clinical practice should ensure and report representation of patients from gender diverse populations who are at risk for health inequities.	Limited to PROMs for Rheumatoid Arthritis.
5	Attitudes toward gender-neutral Spanish: acceptability and adoptability. (2021) *Frontiers in Sociology* [30]	Bonnin, Juan Eduardo; Coronel, Alejandro Anibal	South America, Argentina	A survey conducted to determine attitudes toward gender-neutral language in Argentina, with two hypotheses “1) that favorable acceptance toward gender-neutral language does not imply extensive willingness to use it; 2) that its use is more readily accepted and used when addressing someone, rather than accepting it as a linguistic change.”	4202 participants from general population of Argentina.	Online survey on social networks.	Online survey to explore relevant cases with regard to linguistic attitudes toward non-binary, non- standard linguistic forms.	There is an acceptance in attitudes towards the use of non-binary language, but it does not imply that everyone is committed to using it themselves.	Does not directly address the need to use gender neutral language in generic health questionnaires.
6	Tailored recruitment strategies among transgender and non-transgender veterans: participants’ perceptions and response. (2021) *Military Behavioral Health* [6]	Boyer, Taylor L; Rodriguez, Keri L; Hruska, Kristina L; Klima, Gloria J; Vazzano, Jesse K; Shipherd, Jillian C; Kauth, Michael R; Montgomery, Ann Elizabeth; Blosnich, John R	USA	To determine: 1) what participants thought about recruitment letters that included the word “transgender” in the text; 2) whether transgender participants would mention that inclusion of the word “transgender” facilitated their recruitment; 3) what, if anything, cisgender participants thought about recruitment letters that included the word “transgender” in the text.	100 transgender, 100 cisgender male, and 100 cisgender female US military veterans.	Qualitative study design using interviews with respondents and coding interviews using rapid identification of themes from audio recordings.	Tailored recruitment letter included the word “transgender” and general recruitment letter without the word “transgender”. Telephonic interviews exploring the participants’ thoughts regarding the recruitment letter they received.	Transgender participants were positively supportive of the use of the word “transgender” in the tailored recruitment letter and cisgender recipients were not confused or negative about it. This suggests that trans- gender-related language in recruitment materials is accepted by all participants.	Including the word “transgender” was a positive consideration by cisgender participants, albeit in the context of recruitment letters.
7	Does gender-inclusive language affect psychometric properties of the Illinois Rape Myth Acceptance Scale-Short Form? A two-sample validation study (2022) *Journal of Interpersonal Violence* [31]	Canan, Sasha N; Cozzolino, Lauren; Myers, Jaime L; Jozkowski, Kristen N	USA	To determine the validity of the gender-inclusive language update to the Illinois Rape Myth Acceptance Scale-Short Form (IRMA-SF) in sexually and gender diverse populations.	First data collection sample consisted of 933 cisgender women or transgender individuals who identify as lesbian, bisexual, or heterosexual. The second sample included 1384 cisgender women, transgender individuals, and cisgender men who identified as lesbian/gay, bisexual, and heterosexual.	Online survey on social media.	Illinois Rape Myth Acceptance Scale-Short Form (IRMA-SF)	The modified, gender-inclusive version of the IRMA-SF found that rape myths surrounding alcohol use and intoxication are more readily identified when participants do not have to consider gender as a confounding variable.	The analysis of the article is focused on rape myth acceptance which is not directly useful in this review. However, the discussion of the usefulness of gender-neutral language and how it affects the validity of an outcome instrument could be applied.
8	Does your organization use gender-inclusive forms? Nurses’ confusion about trans* terminology. (2015) *Journal of Clinical Nursing* [32]	Carabez, Rebecca; Pellegrini, Marion; Mankovitz, Andrea; Eliason, Mickey; Scott, Megan	USA, San Francisco	To describe nurses confusion around trans* terminology and to assess LGBT-sensitive nursing practice.	268 undergraduate nursing students	Qualitative research using face to face interviews	The 16-item scripted interview based on the Health Care Equality Index (HEI) addressing institutional policies to ensure quality health care to LGBT patients and families.	Nurses did not know what gender-inclusive meant and tended to confuse sex with gender identity. They were not aware of the relevance of gender-inclusive forms. Assumptions that all clients are heterosexual and identify as either male or female can negatively impact some patients’ health care. Mentions that gender-inclusive forms allow all people to be seen, heard and included in all aspects of health care.	Even though limited to nurses’ interviews, the discussion centres around gender-inclusive forms allow all people to be seen, heard, and included in all aspects of health care.
9	Identifying male victims of partner abuse: a review and critique of screening instruments. (2015) *Partner Abuse* [33]	Chitkara-Barry, Anjuli; Chronister, Krista M	Not applicable - a review	To provide: 1) a review of screening instruments to identify partner abuse victims 2) a gender-inclusive approach to critique key partner abuse screening measures and protocols used currently with men.	Initial literature search yielded 20 measures of partner abuse; 8 met the inclusion criteria	Review and critique	The Revised Conflict Tactics Scale The Computer-Based Intimate Partner Violence Questionnaire (IPV) HITS - assesses the frequency of Partner Abuse in intimate relationships Multidimensional Measure of Emotional Abuse Obsessive Relational Intrusion Ongoing Violence Assessment Tool Partner Violence Screen Victimization Assessment Tool.	All eight of the screening tools assessed used gender-neutral language. A component of gender-inclusive language is the use of gender-neutral terms, to avoid stereotyping a victim or perpetrator’s identity. Using language that all genders can relate to will help improve accurate reporting. It was stated that further research is needed to identify how the format and administration (self-report vs. telephonic report or Interviewer administered) of partner abuse screening instruments affects the accuracy with which victimization is reported. This could be important in our Proxy and Interviewer Administrated forms. A gender-inclusive approach can be used to better understand the different and similar ways in which everyone reports on screening tools.	The article is focused on identifying male victims of partner abuse, however the examples of how gender-neutral language can improve reporting are useful.
10	An exploration of terminology related to sexuality and gender: arguments for standardizing the language (2014) *Social Work in Public Health* [34]	Eliason, Michele J.	USA	“The article explores some of the ways that sex/gender and sexuality terms have been used in health-related research and in keyword searches in the health sciences.”	Not applicable as it was a discussion	A discussion on some research literature	Articles discussing the use of sexuality and gender terminology in the health disciplines.	There is much variability in how sexuality is measured in health studies and what terminology is used. Transgender is often, but not universally, accepted as an umbrella term for those who do not fit societal gender norms. Rarely do health questionnaires or interviews contain questions that would identify people along a transgender continuum, so they are never fully inclusive. By standardizing language used in academic writing in research related to sexuality and gender, the ability to compare across studies increases and improves the likelihood of getting sexuality and gender questions on large-scale population health surveys, improving inclusivity.	One researcher’s discussion on reasons for standardising gendered language in heath research, but not directed at health outcome measures.
11	Organizational strategies and inclusive language to build culturally responsive health care environments for lesbian, gay, bisexual, transgender, and queer people (2021) *Journal of Health Care for the Poor and Underserved* [35]	Goldhammer, Hilary; Smart, Alicia C; Kissock, Laura A; Keuroghlian, Alex S.	USA	“This report shares examples of organizational strategies and inclusive language that can be integrated into standard patient- facing processes, forms, and materials to create culturally responsive health care environments for lesbian, gay, bisexual, transgender, and queer people.”	Aimed at health care organizations	A report	Applicable to medical forms	Whether on demographic forms or and phone scripts it is recommended to remove any language that makes assumptions about gender identity or assumes that all people have a binary gender identity. Importance of correct pronoun use to be fully inclusive and avoiding gendered terms. Recommended to ask local LGBTQ community leaders of diverse races, ethnicities, and ages to review and propose changes to forms and materials for cultural relevancy and understanding.	A generalised report on incorporating key changes to language in processes, form, and materials, to cultivate culturally responsive health care environments for LGBTQ patients. The information would still be applicable to ensuring that our questionnaires are also culturally sensitive to all.
12	Are you…? asking questions on sex with a third category in Germany (2022) *Field Methods* [36]	Hadler, Patricia; Neuert, Cornelia ; Ortmanns, Verena; Stiegler, Angelika	Germany	“Given the ambiguous gender- and sex-related terminology in the German language, and the recent introduction of a third sex category, this article tries to shed some light on the following research questions: 1. Does question wording and the presentation of a third response category impact response behavior and data quality in general population surveys? 2. How is the term “Geschlecht” understood in comparison to more specific terms such as “officially registered sex” and “gender identity? 3. How is the third category ‘divers’ understood?”	1135 panel respondents	Experimental online survey	“Questionnaires with varied question wording, either showing the ambiguous German term “Geschlecht” or more distinct terminologies referring to officially registered sex or gender identity. Further, we varied whether respondents were shown the third category. Understanding of the respective terms was examined by implementing closed- and open- ended probes”.	Adding a non-binary response option for questions on sex/gender does not decrease data quality. However, it appeared that adding a third category such as “divers” as a sex designation, may lead to systematic bias on the part of cisgender males, many of whom gave non substantive answers and showed irritation at the addition.	Even though the sample consisted mainly of cisgender respondents and some cisgender males showed irritation at the addition of a third sex category, the main message that adding non-binary (gender neutral) response options to surveys does not alter data reliability is useful for future development of gender-neutral outcome measures.
13	Inclusion of LGBTQ persons in research related to pregnancy risk: a cognitive interview study (2018) *BMJ Sexual and Reproductive Health* [5]	Ingraham, Natalie; Wingo, Erin; Roberts, Sarah C.M.	USA San Francisco Bay Area of California, Baltimore, Maryland and other cities.	A survey was created with existing sexual orientation and gender identity measures, and new gender-neutral terminology and existing contraception and pregnancy intentions questions that were modified to be gender neutral.	39 individuals aged 18–44 years who were assigned female at birth and identified as LGBTQ.	Two steps - first, developed a survey, then conducted cognitive interviews.	SOGI (Sexual Orientation and Gender Identity) measures were used to assess demographic characteristics, determine study eligibility and to help assess pregnancy risk.	Participants responded positively to gender neutral language. Use gender neutral language to improve cultural sensitivity. The article does advocate for culturally sensitive inclusion LGBTQ individuals in research, by using gender neutral language.	Limitations to generalisability as this was not tested on cisgender heterosexual women and was focussed on abortion and contraception, not general health.
14	Initial evaluation of a gender-inclusive version of the Illinois Rape Myth Acceptance Scale (2021) *Psychology of Sexual Orientation and Gender Diversity* [3]	Johnson, Nicole L; Lipp, Natania S; Stone, Hallie K.	USA	“The study aims to expand understanding of rape myths to the LGBTQ + community through the initial evaluation of a gender- inclusive rape myth acceptance scale.”	575 participants, 325 identified as LGBTQ + and 250 who did not.	Online survey	The modified Illinois Rape Myth Acceptance Scale (IRMAS) was altered to remove gendered language (e.g., he or she) and replaced with gender-inclusive language (e.g., they).	The gender-inclusive version of the modified Illinois Rape Myth Acceptance Scale (IRMAS) showed strong reliability and structural validity in both LGBTQ + and non-LGBTQ + participants. The authors felt that further investigation was needed to determine whether participants read the questionnaire with the same gendered assumption of male perpetrator and female victim, despite gender neutral terms being used. However, they suggested that it was still important to use a gender-inclusive scale, to allow for more flexibility and representation amongst participants.	Even though the focus of the survey was on sexual violence, the finding that inclusive language ensures that those with diverse gender identities to see themselves in the outcome measure, which may increase feelings of belonging and participation.
15	Building culturally sensitive assessments for transgender clients: best practices for instrument development and the adaptation process (2017) *Journal of LGBT Issues in Counselling* [37]	Oberheim, S. Tyler; Swank, Jacqueline M; DePue, M. Kristina	USA	The purpose of the article was to discuss best practices for designing and adapting instruments for the transgender population.	Not applicable as it was a discussion	The article discussed the history of recognizing and advocating for transgender issues within the counselling profession. Best practices for instrument development and adaptation related to the transgender population. Implications of using trans-sensitive assessments in clinical practice and counsellor education.	Not applicable as it was a discussion	Historically, prejudice and stigma has been attached to transgender identities and as such most assessments used heteronormative language. Developers of new assessment instruments or adaptations of instruments used in counselling should ensure that the language and concepts are not uncomfortable to transgender and gender-conforming individuals. “They should use current transgender-affirmative terminology and less gendered language, adhering to participants’ declared or affirmed pronouns/name. Care is needed in wording the items within an instrument and in adapting an existing instrument, professionals must be aware of the importance of revalidating the instrument. In advocating for equality towards all gender minorities it is necessary to obtain experts review of the new or adapted instrument to determine if it is appropriate for all populations.”	While the focus of the discussion is on counselling instruments, the general discussion on best practices for developing or adapting health instruments to ensure gender minorities do not feel excluded is relevant to this review.
16	Considerations for culturally sensitive research with transgender adults: a qualitative analysis (2017) *Journal of LGBT Issues in Counselling* [38]	Staples, JM: Bird, ER: Masters, T: George, WH	USA	“The aim of the study was to provide guidance on conducting culturally sensitive research with transgender participants.”	247 trans participants	A quantitative online survey	Questions investigating relationships among minority stress, sexual behavior, and mental health, followed by four open-ended questions were presented to participants: 1) What was your experience like participating in the Trans* Health Project? 2) What did you like about the survey? 3) What did you dislike about the survey? 4) What suggestions do you have for improving the Trans* Health Project?	Participants reported feeling connected and understood by having their experiences as a trans person validated while completing the survey, rather than feeling ignored as they did by other surveys. They commented positively on the response options and language used which were fully inclusive.	The participants were all transgender persons, but the findings that measures may need to be modified to include less binary language (e.g., using “their” in place of “his/her”) to be fully inclusive of all participants is relevant. Additionally, the suggestion that the trans community who identify as non-binary should read surveys and provide feedback to avoid binary gender identity language, is also important.
17	The dark side of gendered language: The masculine-generic form as a cause for self-report bias (2015) *Psychological Assessment* [16]	Vainapel, Sigal ; Shamir, Opher Y; Tenenbaum, Yulie; Gilam, Gadi	Israel	To determine whether statements written in an incompatible gendered form would bias the other gender’s reports. Hence, women would provide different self-reports when completing a questionnaire written in the masculine-generic form (gender in- compatible) compared with a gender-neutral form (gender compatible).	90 Tel Aviv University students	Questionnaire	Two Hebrew versions of the Motivated Strategies for Learning Questionnaire (MSLQ) were used—a masculine-generic version and a gender-neutral version.	“The differently gendered form of the questionnaire affected men’s and women’s scores differently. Women reported lower scores when filling out the masculine-generic form than they did when filling out the gender- neutral form. Meanwhile, men reported the same score in both types of questionnaire. In the self-report section on in self-efficacy, a difference in scores between women and men was observed in the masculine- generic form, but the difference was reduced significantly in the gender-neutral form. This indicates that to avoid self-report inaccuracies and bias due gendered languages, the gender-neutral form is preferable over the masculine-generic form, which is the format of many gendered languages such as Spanish, German, Hindi, and Hebrew.”	This was not a health outcome measure, but the main message that there is less bias in reporting when using the gender-neutral form, is relevant to this review.
18	Heterocentric language in commonly used measures of social anxiety: recommended alternate wording (2013) *Behavior Therapy* [39]	Weiss, Brandon J; Hope, Debra A; Capozzoli, Michelle C.	USA	“The goal of the study was to develop and test alternate gender-neutral wording on commonly used self-report measures of social anxiety that use heterocentric language.”	405 undergraduate students at the University of Nebraska–Lincoln	5 questionnaires with new gender-neutral items interspersed throughout the measures in a fixed order, and each measure included both original and new items.	5 self-report measures for the assessment of anxiety that contained the wording “opposite sex” or other language that assumes heterosexuality. Social Interaction and Anxiety Scale (SIAS) Interaction Anxiousness Scale (IAS) Social Phobia and Anxiety Inventory (SPAI) Social Avoidance and Distress Scale (SADS) Brief Fear of Negative Evaluation (BFNE).	The study provided some evidence for equivalent or slightly stronger psychometric properties of alternate gender-neutral item wording, compared to heterosexual language. Using gender neutral wording could improve measurement accuracy for sexual minorities as well as heterosexual respondents.	It is not known whether the results would generalize to community or clinical samples. However, it was suggested that individuals who are not exclusively heterosexual, may lead to inaccurate measurement reporting or alienation of these respondents, if a self-report measure that assume heterosexuality, is used, which is useful to this review.

### Collating, summarising and reporting

1. Following data extraction and description of study characteristics, the articles were summarised according to outcomes of interest, target populations and how they addressed the scoping review research questions.

### Populations

Most articles only reported on gender when describing the sociodemographic characteristics of participants. In three articles sex/gender were used interchangeably [25, 28, 36], five identified participants as binary male or female [4, 16, 25, 28, 33], and three were focused exclusively on LGBTQ + participants [5, 37, 38]. Only six articles included cisgender men, cisgender women and diverse gender options [3, 6, 30, 31, 36, 39]. The remaining articles were discussion papers [29, 32, 34, 35], or systematic reviews [25, 33], focused on gender terminology in health care and research. The majority (*n* = 11) of the articles were from the USA [3–6, 37, 38] (32) [32, 34, 35, 39] with only one from Germany [36] and one from Israel [16].

### Degree to which articles addressed the research questions

The first research question was: “What rationale is provided to support the development and use of gender-inclusive language in different countries?” The majority of articles originated from the USA, but it was generally found that there is a need to adopt standardized terms and concepts, and to integrate these into health care and research to ensure inclusivity in health care and consistency across research [28, 34]. However, the complex nature of gender identities makes it difficult to agree on terminology that includes everyone on the gender identity continuum [34]. Using gendered language is well documented as creating possible barriers to health care and exclusion in health research for minority gender groups [25, 28, 29, 32, 34, 35, 37, 38]. Many commonly used demographic questions do not offer gender-neutral options [29]. Having only an “other” option is not sufficient for full gender identification [34, 37].

In relation to the second research question: “What strategies and processes have been used to develop and use gender-inclusive language in different language groups?”, some articles discussed strategies and processes used to develop and use gender-inclusive language in health data collection instruments and measures. For example, a modified version of The Illinois Rape Myth Acceptance – Short Form was developed and validated with gender-inclusive terminology [31]. Specific examples included using gender-neutral terms to replace gendered wording (e.g., “Rape accusations are often used as a way of getting back at men” became “Rape accusations are often used as a way of getting back at someone”) [31]. Others reported using the singular “they” in lieu of “he” or “she” pronouns [40]. Another study, also focused on modifying the Illinois Rape Myth Acceptance Scale to improve inclusivity and representation of LGBTQ + people, used this approach [3]. Using gender-neutral language in surveys was found to increase response rate in a study assessing the impact of gender-neutral language on the disclosure of sexual violence [4].

In addressing the third research question: “What specific examples are reported of gender-inclusive language being applied in health outcome measures?”, a study describing nurses’ confusion around trans terminology, suggested that amending all patient-history and intake forms to be gender-inclusive could improve culturally competent health care [32]. Specific examples they described included adding a ‘domestic partnership’ marital option on forms, in addition to ‘single’, ‘married’, ‘divorced’ or ‘widowed’. They noted that just adding transgender to the male/female question, was not considered sufficient for the range of trans patients. Therefore, they recommended asking ‘What sex were you assigned at birth?’ and ‘What is your current gender identification?’ (with response options, man, woman, trans*, genderqueer/gender nonconforming or other). It was also reported to be useful to identity the pronouns preferred by patients, but no examples of how to achieve this were given.

One study used a gender-inclusive approach to review the literature on eight different self-report partner abuse questionnaires [33]. All eight questionnaires used gender-inclusive language to avoid stereotyping the victim or perpetrator (e.g., referring to the ‘partner or person you are in a relationship with’). Using language that all genders could relate to was found to improve the accuracy of reporting. It was noted that further investigation was warranted into how the mode of administration (e.g., self-report with pen and paper or electronically versus verbal administration via phone or delivered by a clinician), might affect reporting.

In order to include LGBTQ + people in a survey about abortion and contraception care, existing sexual behaviour and reproductive health questions were adapted to make them gender neutral by replacing “man”, “husband”, “boyfriend” with “person”, “spouse” and “romantic partner”. This was reported as improving cultural sensitivity in research studies [5].

An article discussing best practices for developing, adapting, and using assessments with transgender clients, developed a guide to allow for inclusion of the transgender population [37]. The guide emphasised the importance of “being familiar with current transgender-affirmative terminology and being aware of the importance of using the least restrictive gender language that adheres to participants’ declared or affirmed pronouns/names”. Another study also identified the importance of having options to report demographic data related to gender [34]; only providing an “other” option, in addition to the binary ‘male’ or ‘female’, was not sufficient and the guide suggests a place for individuals to include their own response, as terminology is constantly evolving. The guide also discusses the importance of having individuals with expertise on the transgender population, review any adaptations to gendered language.

A study examining trans participants’ experiences in participating in research found participants used a range of gender identity labels (e.g., femme, agender, bigender) and pronouns (e.g., hir/hirs, ne/nem). To ensure inclusivity, the researchers provided guidelines which included not limiting response options, avoiding binary gender identity categories, and using ‘they/them/their’ pronouns when referring to participants [38].

In relation to the fourth research question, “ What are the reported facilitators and barriers faced by developers and users while considering gender inclusivity in developing and using health-related questionnaires and how have these been addressed?” none of the articles reported directly on facilitators or barriers to considering gender inclusivity when developing or using health data collection measures, except for difficulty in agreeing on optimal gender-inclusive terminology for use in questionnaires or surveys [28, 29, 34, 35].

## Discussion

All publications considering the use of gendered language in questionnaires were reviewed, with specific focus on health questionnaires to assist the EuroQoL group in developing an appropriate gender-inclusive proxy HRQoL questionnaire. In general, most publications highlighted gaps in representation and advocated for gender-inclusive/neutral language when collecting data in questionnaires There appears to be little focus on this topic outside questionnaires developed specifically for use with gender diverse communities [5, 37, 38] or collecting data on specific phenomenon such as interpersonal or sexual violence [3, 4, 31, 33].

Eight articles discussed adverse consequences of binary gendered language use including exclusion from health care or health research among gender minority groups [25, 28, 29, 32, 34, 35, 37, 38]. The lack of representation on health forms and questionnaires points to inadequate attention being given to the health needs and interests of minority gender groups, which should be an important consideration for developers of questionnaires. Reliability and validity of outcome measures is improved with the use of gender-neutral or gender-inclusive language as was reported in five articles [3, 16, 31, 36, 39]; wording was clear and understood by all. Response rates were shown to improve with the use of gender-inclusive language [4, 33]. Non-binary response options also improved internal consistency in a gender-neutral Spanish survey [30]. Good acceptance of gender-inclusive language by all participants was reported in three articles [5, 6, 30], indicating that this would not be objected to in some cultures. Only one paper found that adding non-binary gender response options (e.g. ‘gender diverse’) led to irritation on the part of cisgender males, but they also found such inclusion did not reduce data quality overall [36]. Using inclusive language that all genders can relate to, and avoiding stereotyping, can improve the accuracy of self-reported data [33]. It appears that advantages of gender-inclusive language outweigh potential disadvantages.

Only one paper discussed gaps in representation if diverse gender identities were not considered when developing PROMs. This study focussed on PROMs instruments for Rheumatoid Arthritis and found that “no studies examined the impacts of diversity in gender identity and expression nor sexual orientation on PROMs” [25]. The other 17 articles in our scoping review were focused on assessing specific outcomes for LGBTQIA+/trans groups or surveys for the general populations [3–6, 16, 28–39]. Surprisingly, despite several articles reporting the need for longer lists of gender response options, no paper was identified in the scoping review which explicitly and directly examined preferred gender-inclusive linguistic options to include in health instruments. It is also important to note, that a potential adverse consequence of the use of specific gendered categories was reported in one article, where transgender individuals’ expressed concern that they may be “outed” as a consequence of research using specific gendered categories [6]. Such concern is manifest internationally, where some countries have laws or societal behaviours which actively discriminate against (and even criminalise) non-binary gendered individuals. However, three English-language articles reported that the concept of “preferred pronouns” and singular “they” were found to be readily acceptable to express non-binary gender identities [41–43].

There are some health questionnaires developed for proxy completion (e.g., DEMQOL-Proxy-U [44] KINDL Proxy version [45] and PedsQl Proxy [46]), but no literature was found in our scoping review which discussed the use of gendered language used in these questionnaires. Some other instrument developers simply ask proxies to complete the same ‘self-complete’ version of the questionnaire (rather than positioning the proxy in relation to the third person) [47, 48] which avoids gendered language and may a reason for the lack of focus on this in the peer-reviewed issue. As mentioned previously, the EuroQol Group has available HRQoL questionnaires, for completion by proxy respondents, which currently use binary gendered language to refer to the person whose health is being enquired about .

In some countries, the use of gender-inclusive language is highly contested, and there have been instances where it has been considered a linguistic aberration [30]. In Argentina this led to a debate as to whether inclusive language should be prohibited or not [30]. A survey undertaken in Argentina sought to understand people’s attitudes towards gender-neutral terminology. It was found that even though some were accepting of gender-neutral language, it was not something they were personally willing to adopt [30]. In Germany, the only legal and medical definition by which all non-binary people are categorised is ‘gender diverse’ which fundamentally denies the identities of trans and ‘other’ gender diverse groups [49]. In France, debates about identity politics and the use of inclusive language have been seen by some as a threat to national unity [50]. Worryingly, as mentioned, the collection of specific gender-inclusive information in health questionnaires may even expose non-binary gendered respondents to risk [51]. Others have observed, “Calls for greater representation of marginalised groups often equate visibility with acceptance and empowerment. But visibility is not a blanket good. Being visible, for trans people, is replete with complexities and ambivalences (Koch-Rein et al., 2020), depending largely on how they are visible, and to whom” [52, 53]. Safety and participation for everyone is a right which should not require survey participants to have to ask to be included [54]. However, aside from the complexities associated with grammatically gendered languages, societal and political attitudes towards LGBTQIA+/trans people may clearly affect the development and use of gender-inclusive health questionnaires.

In terms of strengths and limitations associated with the scoping review, it is arguably a strength that the review has identified the need for an increased focus on gender-inclusiveness when developing health questionnaires. A limitation of the scoping review was there were no established MeSH terms, used in MEDLINE/PubMed, to help identify literature related to gender-inclusiveness. This makes it likely that some eligible articles have not been identified. Further, despite searching eligible articles in three other languages, only eligible English language articles were found, and, while it was clear that gender-inclusive language was acceptable to a broad range of English respondents, the complexities associated with gender-inclusive wording in other languages (or other language families) has not been able to be explored.

Findings of this scoping review point to the importance of all those developing health and HRQoL instruments considering the ethical imperatives of ensuring all potential respondents, including LGBTQIA + broadly, and trans people, specifically, can recognise that the instrument is intended to include them; thereby facilitating their participation (13) [14, 26, 28, 37, 55]). Adopting gender-inclusive language in questionnaires clearly reduces the risk of excluding gender minority groups [15, 56]. Although the scoping review found few specific examples of how others have used gender-inclusive language in health questionnaires, many strengths of gender-inclusive language usage were identified. As binary gendered language is currently used in some EuroQoL ‘source’ proxy versions, it is recommended that gender-inclusive proxy versions be developed (e.g. using singular ‘they/their/themselves’). While the initial focus may be on developing gender-inclusive English language proxy versions, this should be accompanied by testing the acceptability and translatability of gender-inclusive wording into other languages.
